# Supplementary material for: Are carbon emissions trading and green financial instruments synergistic? -Comprehensive quantitative research based on content analysis
Source: PLoS One. 2024 Mar 7;19(3):e0298601. doi: 10.1371/journal.pone.0298601 (PMC10919601; doi:10.1371/journal.pone.0298601)
Supplement: S1 Data — (ZIP) [file pone.0298601.s001.zip › Data/地级市Z_F工作报告.zip!/░▓╗╒/┴∙░▓╩╨/└┤╫╘╣1⁄2╓┌║┼í╛┬φ┐╦╩2╛▌═°í┐_2005.docx]

各位代表：
　　我代表市人民政府，向大会作政府工作报告，请予审议，并请市政协委员和其他列席人员提出意见。

**一、过去五年工作回顾**

　　撤地设市五年来，全市人民在省委、省政府和市委的坚强领导下，高举邓小平理论和“三个代表”重要思想伟大旗帜，认真贯彻党的十六大和十六届三中、四中全会精神，围绕加快发展、富民强市、全面建设小康六安，开拓创新，团结奋斗，胜利完成了市一届人大一次会议确定的目标任务。 
　　**国民经济稳步发展，综合实力逐步壮大。**全市生产总值年均增长7.9%，2004年达到253.1亿元。经济结构发生积极变化，三次产业比重为31.6:34.7:33.7。农业基础地位加强，农业总产值年均增长3.2%，达到149.4亿元。工业速度效益明显提升，规模企业增加值年均增长9.7%，达到34.6亿元，效益综合指数由1999年的94.5提高到121.6。第三产业加速发展，社会消费品零售总额年均增长7.7%，达到99.7亿元，旅游业、信息业、物流业等新兴产业发展势头强劲。民营经济占生产总值比重达到48%，民营企业由1999年末的1221户发展到3696户、注册资金23.6亿元。财政收入同口径年均增长9.2%，达到16.1亿元。
　　**基础设施建设强力推进，发展后劲明显增强。**累计完成全社会固定资产投资306.5亿元，年均增长14.3%；争取各类政策性资金110亿元。宁西铁路六安段建成通车，商景、合西、合阜高速公路六安段开工建设，市到县（区）区间快速通道基本形成。中直大型粮库及铁路专用线建成运营。临淮岗主体工程基本建成，白莲崖水库辅助工程开工，城西湖蓄洪大堤除险加固等工程顺利实施，移民迁建和保庄圩建设积极推进。江淮分水岭综合治理开发取得阶段性成果。城乡电网改造基本完成并实现居民生活用电同网同价。通信设施建设成效显著。全面启动“568”计划，累计开工项目172个、完成投资132亿元。
　　**中心城市建设日新月异，城镇面貌发生重大变化。**完成了六安城市总体规划修编。累计投入67亿元，新建改建城区干道67.5公里，改造旧城1.83平方公里，开发商住房面积260万平方米。环城路、火车站广场、滨河公园、中心广场、广电中心等重点工程相继竣工，“七纵七横连三环、两带三区四广场”的中心城市框架基本形成，城市面貌发生根本性改观，实现了“三年打基础、五年见成效”目标。县城和集镇建设全面推进，全市城镇化率逐年提高。
　　**各项改革不断深化，对外开放成效明显。**国有工业企业改制退位基本完成。农村税费改革扎实推进，各项惠农政策全面落实，农民负担大幅减轻。政府机构改革稳步实施，乡镇区划调整顺利完成。公共财政改革力度加大，医疗、住房等改革继续深化。招商引资取得历史性突破，累计到位境内资金90.7亿元、境外资金1.3亿美元，华电六安电厂、长江精工、西都时代广场、安徽商之都六安商厦等一批重点项目落户我市。开发区和各县（区）工业园区建设力度加大。对外贸易连续保持全省较好位次，累计完成进出口总额4.6亿美元，年均增长16.2%。对外交流与合作日益扩大，与新疆巴州、江西吉安市、芬兰瓦尔考斯市结为友好城市。
　　**人民生活水平逐步提高，社会保障体系建设全面推进。**城镇居民人均可支配收入和农民人均纯收入分别年均增长7.7% 和3.4%，达到6103元和2117元；城乡居民储蓄存款余额173.4亿元，比1999年末增加101亿元。累计新增就业岗位11.6万个，城镇登记失业率控制在4.1%。劳务经济成效明显，年输出140万劳动力、创收50亿元以上。国有企业下岗职工基本生活费、企业离退休人员养老金按时足额发放。涵盖养老、医疗、失业、工伤等险种的社会保险体系初步建立。城市低保实现应保尽保。农村特困救助全面启动。民工维权扎实有效。生产救灾成绩突出。累计投入扶贫资金12.5亿元、脱贫106万人。
　　**社会事业全面发展，民主法制和精神文明建设不断加强。**教育“两基”成果得到巩固，以县为主的义务教育管理体制改革深入推进，农村中小学危房改造一期任务全面完成。皖西学院、六安职业技术学院和安徽国防科技职业学院组建升格，在校生规模分别达到11000人、5000人和3500人。人口、资源、环境三项基本国策进一步落实，人口出生率控制在12.7‰。科技和人才工作不断加强， 专家大院建设和科技特派员创业行动启动实施。城乡医疗卫生条件有所改善，抗击非典和防治高致病性禽流感夺取胜利。文化、广电、新闻出版业健康发展，人民群众精神文化生活不断丰富。全民健身运动深入推进，市首运会成功举办。各类群众性文明创建活动蓬勃开展，“四五”普法和创建“三型机关”活动扎实有效。政府依法行政得到加强。市政务服务中心“一站式”服务启动。市政府建立了定期向人大常委会报告工作和向政协通报工作制度，认真办理人大议案、代表建议和政协建议案、委员提案。安全生产各项措施进一步落实，社会治安综合治理全面推进，信访工作得到加强，社会秩序保持稳定。国防后备力量建设得到加强，被省授予双拥模范城。审计、统计、档案、保密、地方志、地震、气象、对台、外事侨务、民族宗教、人防、妇女儿童、老龄和残疾人等各项工作都取得了新成绩。
　　2004年，全市主要经济指标完成情况为近年来最好水平。生产总值比上年增长14%，是1998年以来首次两位数增长；财政收入增长10.7%；招商到位境内资金40亿元、境外资金4408万美元，分别增长58%和40.6%；全社会固定资产投资增长29.3%；社会消费品零售总额增长11.9%；进出口总额增长29.7 %；城镇居民人均可支配收入、农民人均纯收入分别增长18.8%和21.9%。
　　各位代表，过去五年是我市国民经济实现较快发展、改革开放迈出重大步伐、城乡面貌发生最大变化、人民群众得到更多实惠的五年。五年来，我们始终坚持把解放思想与实事求是结合起来、创新精神与务实作风结合起来，探索出符合六安实际的发展路子；始终坚持在改革中增创体制优势，在开放中拓展发展空间；始终坚持重点突破、整体推进，集中力量解决事关六安发展全局和未来的重大问题；始终坚持以民为本，注重维护好、实现好、发展好人民群众的根本利益；始终坚持“三个文明”协调推进，为全市加快发展提供强大精神动力和智力支持，使全市人民保持奋发有为、昂扬向上的精神风貌。五年来，我们走过了不平凡的历程，取得了来之不易的成就。这是在市委的正确领导下，全市人民团结拼搏、开拓进取的结果，同时，也得益于原地委、行署多年来奠定的工作基础。这些我们都将倍加珍惜。
　　我们还清醒地看到，在经济社会发展中，还存在不少困难和问题。主要是：经济总量不大，人均水平较低，综合实力不强；城乡居民生活整体水平不高，部分群众生活还比较困难；经济结构不优，工业化、城镇化、经济外向度水平低的状况还没有根本改变；基础设施建设相对滞后，农业抗灾能力仍较薄弱；财政收支矛盾较为突出，乡村债务化解缓慢；发展环境有待进一步优化；影响稳定的因素依然存在等。对此，一定要高度重视并采取切实措施认真加以解决。

**二、今后五年基本任务**

　　今后五年，是全面建设小康六安、实现跨越式发展、推进战略性崛起的关键时期。刚刚胜利闭幕的市第二次党代会，提出了“三大跨越”的历史任务，为全市人民指明了奋斗方向。我们必须紧紧抓住中央促进“中部崛起”和加大“三农”扶持力度的政策机遇，充分把握沿海发达地区加速产业转移的有利条件，善于发挥正在实施的四条高速公路和两条铁路彰显的区位优势，在区域经济竞争日益加剧的新形势下，扬长避短，开拓奋进，努力把六安改革开放和现代化建设的伟大事业推向新阶段！
　　根据市第二次党代会部署，今后五年政府工作总体要求是，**高举邓小平理论和“三个代表”重要思想伟大旗帜，认真贯彻党的十六大和十六届三中、四中全会精神，坚持以人为本，以科学发展观为指导，以抢抓机遇、乘势而上、奋力崛起为指引，以“568”计划为主抓手，坚持招商引资主战略和工业化核心战略，坚持基础设施建设先行和城乡统筹，以适度超前的城镇化，带动相对滞后的工业化，促进农业产业化，加速信息化，推动体制、结构、科技、环境和管理创新，促进物质文明、政治文明、精神文明协调发展，全力实现“三大跨越”，为全面建设小康六安奠定更加坚实的基础。**
　　为此，我们要确保全市生产总值年均增长11%以上，2009年力争比2004年翻一番，财政收入同步增长，实现综合经济实力新跨越；我们要确保城镇居民人均可支配收入和农民人均纯收入分别年均增长8%和6%以上，2009年达到10000元和3000元，实现人民生活水平新跨越；我们要确保中心城市基本形成60平方公里框架、近50万人口规模，大力实施治水安民工程，实现中心城市建设和农业抗灾能力新跨越，把六安建成功能齐备、设施完善、产业集聚、辐射带动能力较强的区域中心城市。
　　**（一）加快工业化进程。**坚持把工业经济放在核心位置，确保规模工业增加值年均增长16%，二产占生产总值比重达到40%以上，基本形成工业主导型经济增长格局。依托骨干企业，实施一批牵动性强、关联度大、产业链条长的重点项目，培育食品及农产品加工、机械制造、纺织服装、医药化工、建筑建材、冶金、能源等重点产业。实施“规模倍扩、效益倍增”工程，促进重点企业扩张，力争培育年销售收入超50亿元企业1家、超10亿元企业3-5家、超5亿元企业6-10家、超亿元企业40家以上。注重运用高新技术改造传统产业。推进名牌战略，培育3-5个“中国驰名商标”、“中国名牌产品”和40个左右省著名商标。扶持3-5户成长性好的企业上市。努力把市开发区建成工业城，引导工业园区向专业化、特色化方向发展。 
　　**（二）加快农业产业化进程。**进一步强化农业基础地位，坚持以工业的理念抓农业，以产业化经营为突破口，提高农业综合生产能力。合理调整优势农产品区域布局，逐步壮大优质粮油、畜牧、水产等产业规模。培育一批带动力强的龙头企业和企业集团。发展各类中介组织，组建壮大各级产业协会。建立健全市场营销体系，重点发展50个农产品市场，建成3-5个年交易额5亿元以上的大型专业市场。
　　**（三）加快城镇化进程。**强力推进城镇化战略，加速各类生产要素向城镇聚集，确保城镇化率每年提高2个百分点以上。加大中心城市建设力度，按照完善功能、提升品位、建设生态城市的要求，健全城市规划体系，坚持市区联动，完成旧城改造，加快新区开发，建成道路主干网，完善市政设施，强化城市产业支撑力和承载力。提高县城建设水平和载体功能，建设特色小城镇，发展一般集镇，引导城镇走集约化发展道路。
　　**（四）加快经济外向化进程。**大力实施招商引资主战略，确保到位境内资金和引进境外资金年均增长15%以上。推动建立国际友城，加强对外合作交流，提高利用外资水平。抓住沿海地区产业梯度转移的重大机遇，提升承接能力，力争把六安建成皖西重要的经济板块。坚持以优势骨干企业为依托，积极寻求与国内外大企业、大集团合资合作。不断扩大出口贸易，进出口总额年均增长15%以上。积极探索在商业、旅游、卫生、文化、教育等领域利用市外资金途径。
　　**（五）加快市场化进程。**坚持社会主义市场经济取向的改革，推动经济成份多样化、投资主体多元化、运行机制市场化。完善和落实政策措施，放手发展非公有制经济。深化投资体制改革，全面推行企业投资项目核准制和备案制。不断提升传统市场，积极发展产权、资本、土地、技术和劳动力市场，促进生产要素有序流动、合理配置。扎实推进以政府信用为先导、企业信用为重点、个人信用为基础的信用六安建设，创造良好的市场环境。
　　**（六）加快社会和谐和文明化进程。**按照“民主法治、公平正义、诚信友爱、充满活力、安定有序、人与自然和谐相处”的要求，着力构建和谐六安。坚持高度关注民生，统筹发展各项社会事业，不断提高社会文明程度。积极推进民主法制建设，保障人民群众依法行使民主权利。大力开展群众性文明创建活动，争创全省文明城市。深入开展全民国防教育，加强国防后备力量建设，做好拥军优属工作，争创全国双拥模范城。深入实施科教兴市和人才强市战略，建立和完善科技创新服务体系，推进数字六安建设，实施新一轮教育振兴行动计划，促进人口大市向人力资源大市转变。积极发展文化事业，重点开发皋陶文化、寿春楚文化、红色文化等资源，打造六安文化品牌。建立健全医疗服务和全民健身体系。坚持在发展中解决就业问题，累计新增城镇就业12万人以上，城镇登记失业率控制在4.5%以内。积极改善困难群众生产生活条件，全面完成扶贫攻坚任务，探索建立农村最低生活保障制度，完善社会保障体系。增强可持续发展能力，进一步稳定低生育水平，保护青山绿水，推动六安走生产发展、生活富裕、生态良好的发展之路。
　　各位代表，六安经济社会加速发展的态势已经显现，全市上下解放思想、奋力创业的氛围日益浓厚。只要坚定信心，团结拼搏，我们的目标一定能够实现，六安的未来一定更加美好!

**三、2005年主要工作**

　　今年是全面完成“十五”计划、衔接“十一五”发展的重要一年，也是新一届市政府的开局之年。因此，我们必须确保经济发展有一个较快速度和较高质量。建议主要预期目标是：生产总值增长11%；财政收入同口径增长16.3%；全社会固定资产投资增长20%；社会消费品零售总额增长12%；进出口总额增长20%；新增城镇就业2.35万人以上，城镇登记失业率控制在4.3%以内；城镇居民人均可支配收入增长8%；农民人均纯收入增长6%；人口出生率控制在12.69‰以内。
　　**（一）加快农业和农村经济结构调整，促进农民增收。**坚持关注农村、关心农业、关爱农民，推动农业和农村经济发展。
　　**认真落实各项扶持政策。**坚持把中央和省出台的各项惠农政策不折不扣地落到实处。从今年起，全部免征农业税，全市农民减负2亿元、人均37.2元；积极争取各项支农补贴资金，全市农民政策性增收2.5亿元、人均43.9元。积极推进财政面向农民补贴资金管理和支付方式改革，实行“指标统一下、资金一户管、补助一卡发、服务一站办、收支一本账”。继续清理涉农行政事业性收费和经营服务性收费，强化农民负担监管。
　　**调整优化农业结构。**加强农业综合能力建设，规划并建设优势明显、各具特色的生产基地，促进优势农产品向优势产区集中，重点建设山区林茶药果、江淮丘岗草食畜禽、平原圩畈优质粮油等12个优势产业带。加快发展名、特、优、稀等市场前景广阔的农产品生产。实施“粮变肉”工程，积极推进畜牧水产业规模化生产。推广优良品种，发展优质专用粮食、双低油菜和优质高效经济作物，提高农产品优质率。
　　**发展农业产业化经营。**围绕“扶持大龙头、建立大基地、开拓大市场、搞好大服务”，提高产业化水平。进一步壮大龙头企业规模，争创省级以上龙头企业10家，培育和认证市级龙头企业30家。大力发展订单农业，鼓励有条件的龙头企业自建生产基地。规范提高现有10个产业协会，组建奶牛等新的产业协会，大力发展农村经纪人队伍。建立和拓宽营销体系，积极参加各类展示展销活动，畅通鲜活农产品运输“绿色通道”。大力发展品牌农业，促进优质农产品进入大中城市市场。积极引导和推进外商资本、民间资本和工商资本投入农业开发。
　　**提高劳务经济水平。**健全劳动力市场信息网络，发展劳务中介组织，扩大劳务输出渠道，拓展境外劳务输出。大力发展农村二三产业，促进劳动力就地转移。推进农村劳动力转移培训“阳光工程”和“绿色证书工程”，举办各种形式的专业培训、订单培训和系统培训，促进技能性就业，逐步把我市建成特色鲜明、层次较高的劳务基地。
　　**强化“三农”服务工作。**加强农田水利、“六小工程”建设，深入推进江淮分水岭综合治理开发。加快农业机械化进程。加强农技推广体系建设，实施农业科技入户工程，抓好科技富民强县试点工作。加强农网建设，强化信息服务。依法管理农资市场，开展农资产品专项整治，确保农资供应。建立健全动物疫病防治体系，高度重视高致病性禽流感防控工作。推进农业标准化，加强农产品质量管理。扩大农户小额信贷，财政新增事业经费重点用于农村发展。 
　　**（二）全面深化企业改革，提升工业经济实力。**深入推进“发展工业学霍山”活动，营造工业发展氛围，确保规模工业增加值增长16%。
　　**加大企业改制力度。**进一步做好部分企业改制扫尾工作，全面完成改制退位任务，努力实现国有实际资产不流失、职工合法权益不受损、投资者合法收益有保障的“三方共赢”目标。
　　**加快重点项目建设。**编制主导产业发展规划，重点实施华电六安电厂2×13.5万千瓦发电机组、长江精工紧固件、华源纺织差别化纤维面料、江汽齿轮系列汽车变速器等10个投资超5000万元的续建项目，加速启动海螺集团110万吨水泥粉磨站、华源长富液体制剂基地、应流集团2万吨铸钢、世林集团大功率节能灯、金安钢铁公司三期技改等10个投资超5000万元的新建项目，努力推进华电六安电厂2×60万千瓦发电机组、霍邱年产200万吨钢铁、霍山无石棉刹车片、星星轻纺出口基地等后备项目前期工作。
　　**做大做强骨干企业。**重点支持迎驾集团、华源纺织、华源药业、华润龙津、江汽齿轮等企业发展，做好长江精工配股增发、世林照明争取上市和星星轻纺上市前期工作，推动应流铸造境外上市，培育一批主业集中、主导产品突出的骨干企业。新增销售收入超5亿元企业2家、超亿元企业6家。
　　**实施中小企业促进工程。**组建管理机构，从创业扶持、技术创新、市场开拓、社会服务等方面为中小企业创造良好发展环境。扶持技术含量高、产品有市场的中小企业快速成长。鼓励中小企业与大企业协作配套，向“专、精、特、新”方向发展，尽快兴起一批小而精、小而强、小而专、小而优的中小企业群。
　　**改善工业经济运行环境。**建立完善重点企业联系制度和服务“直通车”制度。着力构建中小企业担保体系，加快组建各级担保机构。强化银企对接，推进银企合作。
　　**（三）加大招商引资力度，全方位扩大对外开放。**突出招商重点，创新招商策略，打造投资平台，确保到位境内资金和境外资金分别增长20%和15%以上。
　　**提升招商引资规模和质量。**突出对长三角、珠三角和港澳台等重点地区的招商攻关，建立若干区域协作城市。认真筹备参加2005国际徽商大会、第九届中国投资贸易洽谈会等重大招商活动，力求在大企业、大项目合资合作和直接利用外资上取得突破。积极争取世行、亚行贷款，提高间接利用外资水平。
　　**创新招商方式。**在坚持全民招商、会展招商、小分队招商等有效方式的同时，促进招商工作走向专业化、规范化。突出产业招商，实行一个产业、一批项目、一个班子、一笔经费、一抓到底。加强企业招商，发挥企业招商主体作用。抓好招商项目后续工作，努力做到引进一个、办好一个，办好一个、带来一批。进一步改善政策环境，优化服务环境，保障用地环境，营造金融环境，努力形成“投资者是上帝，引资者是功臣，服务者是榜样，阻碍者是罪人”和“人人都创招商环境，大家都树六安形象”的良好氛围。
　　**切实办好开发区、试验区和工业园区。**加大开发区和工业园区基础建设和项目引进工作力度，市开发区要力争完成引资4.5亿元以上，各县区工业园区力争引进投资规模3000万元以上项目不少于3个、完成投资不少于1亿元。各类园区要进一步建立健全公共服务网络，完善政策服务体系，落实投资强度标准，提高土地利用效益。继续推进“工业向园区集中，人口向集镇集中，住宅向小区集中”。积极做好叶集、卅铺、姚李等集体建设用地流转试点，缓解建设用地矛盾。发挥叶集试验区政策和体制优势，打造我市西部发展平台。 
　　**扩大对外贸易。**组织企业积极参加各种出口交易会和专业博览会，加大国际市场开拓力度。优化出口商品结构，促进加工贸易转型升级，鼓励企业使用本地原辅材料和收购本地产品做好出口贸易。
　　**（四）大力推进中心城市建设，进一步改善城乡基础设施状况。**
　　**加大中心城市规划建设管理力度。**认真做好城市专项规划和控制性详规编制工作。多渠道筹集资金建设重点工程。建成烈士陵园及人民广场、市行政中心等在建工程，完成汽车客运站场搬迁任务。加快古城广场及风貌整治等在建工程进度。开工建设长安北路、佛子岭西路大桥等路桥工程和体育馆、皋陶陵园、老年活动中心等重点设施，改造皖西路、大别山西路、皋城西路和健康路及20条社区支路，开展大雁河综合治理，改造常青路步行街，新建改建垃圾填埋场、20座公厕、垃圾中转站、污水管网三期和医疗废物集中处置中心，建成22万平方米居民安置房。进一步理顺城市管理体制，提升城市综合管理水平，集中整治脏乱差，强化物业管理，加快地名标准化建设，推进城市绿化、亮化、美化，不断提高城市的美誉度。
　　**抓好县城和集镇规划建设。**按照“扩大规模、完善功能、美化环境、提高品位”的要求，加强县城规划管理，加快县城建设步伐。依托资源和产业优势，发展一批工矿型、商贸型、旅游型、交通枢纽型和农业产业化型等特色城镇。深化户籍制度改革，引导农民向城镇转移。
　　**加快建设重点基础工程。**积极支持沪汉蓉高速铁路建设，配合做好阜六铁路前期工作；推进商景、合西、合阜高速公路六安段建设，争取开工建设六武高速公路六安段；完成338公里国省道和重要县道新建改建任务，启动874公里农村公路国债项目和通达工程，抓好金寨全国农村公路示范和全省通村公路试点，改造重点旅游景区低等级道路。建成临淮岗工程，完善城西湖蓄洪大堤、正南淮堤、临王段、史河堤防加固等重点治淮工程，加快白莲崖水库、安丰塘除险加固、内河治理和城区防洪及移民迁建等工程进度，开工建设郑家湾电站。建设六安中心11万伏变电所。改善山区和边远地区通信状况。
　　**（五）大力发展服务业，提升三产整体水平。**
　　**增强旅游业综合能力。**促成国家大别山旅游规划编制工作，推进红色旅游工程、绿色生态工程和古文化旅游工程，重点实施国家“红色旅游区”项目，积极申报大别山（六安）国家地质公园，发展各具特色的人文游、生态游、休闲度假游。加强与“两山一湖”旅游网的对接，统筹建设市内旅游网络。围绕“吃、住、行、游、购、娱”，做好系列配套文章。推进旅游景点经营权转让，提升景点层次。加强旅游市场监管，提高旅游业队伍素质。组织争创全省旅游经济强县和优秀农家乐旅游区活动。
　　**提升服务业层次。**大力发展商贸流通、餐饮等劳动密集型服务业，加快发展现代物流，推广连锁经营等新型业态。规划开发城南市场带，建设六安国际汽车城。积极发展资本、人才、技术等生产要素市场，发展会计、评估等中介服务组织和代理、融资等生产服务业，推进家政、社会化养老等社区服务业发展。全面完成国有商业企业改制任务，保护和规范发展一批商贸龙头企业。
　　**促进消费需求增长。**积极培育网络教育、文化娱乐、医疗保健、体育健身等新消费热点。进一步培育放活房地产二三级市场，健全完善住房公积金制度。加强农村流通设施建设，扩大农村消费。发展消费信贷， 提高居民购买力。改善消费环境，维护消费者合法权益。
　　**扎实做好财税金融工作。**强化公共财政职能，进一步完善国库管理制度，深化财政管理体制改革，抓好增收节支工作。充分发挥财政职能，支持财源建设。强化税收征管，改善税收环境。优化财政支出结构，规范非税收入管理，全面实行部门综合预算。进一步加大国债、中央预算内资金等各类政策性资金争取力度。支持国有商业银行改革，努力增加有效信贷投入。加快组建城市商业银行，推进农村信用社改革。继续争取和利用好国家开行的城市建设、扶贫和中小企业项目贷款。积极发展证券、保险业。建立金融协调机制，防范和化解金融风险。
　　**（六）放手发展民营经济，努力壮大县域经济。**鼓励全民创业，坚持以民营经济为主体，扩大总量，提升素质，促进县域经济上台阶。
　　**大力发展民营经济。**进一步解放思想，激发全民创业热情，扩大社会投资规模。制定激励措施，改善创业环境，积极引导机关和事业单位分流人员、下岗职工、大中专毕业生、复退军人和外出务工经商人员，开展各种创业行动。支持社会资本参与城市基础设施建设及社会公益事业发展，进入国家放宽的垄断行业和领域。鼓励民营企业加快发展，壮大规模，增强实力。引导民营企业技术改造，开展创牌行动。从市场准入、项目提供、人才输送、科技支持、信息和金融服务、权益维护等方面为民营企业发展创造良好环境。培养适应市场化要求的民营企业家队伍。引导组建民营企业集团10家以上，民营企业总户数突破4500户。 
　　**加快发展县域经济。**支持各县区在科学规划基础上，整合和开发特色资源，培育优势支柱产业，做大做强重点企业，带动发展中小企业，实现产业连锁和规模扩张。积极发展镇域经济和区街经济。实施县乡财政振兴工程，巩固乡财县管成果，逐步建立科学规范的政府债务预警和偿债机制，缓解县区财政困难，减轻乡村债务，多方筹集发展资金。鼓励各地大胆探索县域经济发展新路子。
　　**（七）加强政治文明和精神文明建设，促进经济社会协调发展。**
　　**进一步加强民主法制建设。**坚持依法治市，做好“四五”普法检查验收工作。推进和规范政务公开、厂务公开、村务公开，认真组织第六届村民委员会选举。合理规划社区布局，促进社区机构、人员、场地、职责落实，完善社区功能，搞好社区服务。注重研究和解决基层民主政治建设中的问题。加强法律援助和民工维权工作，保障弱势群体合法权益。支持工会、共青团、妇联、科协、文联、残联工作。加强国防后备力量建设和兵役工作，重视人防建设，广泛开展双拥活动。
　　**大力推进精神文明建设。**争创全省创建文明城市工作先进市，提高各类群众性文明创建水平，提升市民素质。加强和改进未成年人思想道德建设，培育“四有”新人。积极开展“四进社区”和“三下乡”活动，形成健康向上、文明和谐的社会环境。加大殡改力度，倡导文明风气。  
　　**深入实施科教兴市战略和人才强市战略。**加快建设科技服务体系，推进科普工作，认真开展专家大院建设和科技特派员创业行动，推动实施一批校企合作项目和国家、省级研发项目。进一步巩固教育“两基”成果，加快中小学布局调整，改造农村中小学危房20万平方米，鼓励支持社会力量办学，大力发展职业教育和高中教育。支持皖西学院、六安职业技术学院和安徽国防科技职业学院发展。把人才工作纳入国民经济和社会发展总体规划，设立市人才基金，积极引进人才和国外智力，加强人才培养，壮大人才队伍。推进农科教统筹，促进农科教结合。
　　**加快生态市建设。**以“生态六安”为目标，围绕建设节约型社会的目标，深入开展倡导绿色文明、创建绿色家园活动，增强全社会生态意识。大力发展生态经济，加强国家级生态示范区建设，推进生态家园富民工程，筹办市生态示范农场，发展循环经济型企业。积极调整林业布局，加快发展林业产业，切实保护森林资源。全面开展城市环境综合整治、重点区域和淮河、巢湖流域污染防治工作。抓好土地、矿产、能源及重要原材料节约和综合利用。
　　**加强人口与计划生育工作。**强化农村和流动人口计划生育管理，进一步稳定低生育水平。认真落实农村计划生育家庭奖励政策。推进“关爱女孩工程”和“春蕾计划”。加大案件查处力度，着力解决出生人口性别比失衡问题。
　　**积极发展社会事业。**繁荣文化事业，发展文化产业，深化文化体制改革，大力加强文化设施建设，着力培育文化产业实体，积极发展文艺演出业、文化旅游业、文化娱乐业和艺术培训业，规范网吧经营，保护和使用正版软件，公布首批市级文物保护单位。启动市第二轮修志工作。继续推进农村广播电视村村通工程，不断提高农村广播电视有效覆盖率。加强疾病预防控制体系、重大疫情信息网络体系和卫生执法监督体系建设，建立结核病和艾滋病等重大传染病防治长效机制。抓好农村新型合作医疗试点，加强乡村卫生设施建设。开展国民体质测试，积极备战省体育常规赛。进一步做好统计、档案、外事侨务、民族宗教、地震、气象、对台、关心下一代等工作，重视发展老龄、妇女儿童和残疾人事业。
　　**（八）切实关心群众生活，着力构建和谐社会。**坚持以民为本，始终把人民群众利益放在首位，多做暖人心、稳人心、得人心的工作，努力使人民群众在改革发展中得到更多实惠。 
　　**深入推进就业再就业工作。**就业是民生之本。坚持把促进就业作为经济社会协调发展和构建和谐社会的重要目标，坚持劳动者自主择业、市场调节就业和政府促进就业的方针，实现兴办企业和增加就业的良性互动，确保再就业 1.22万人以上。加强街道、社区劳动保障平台建设，积极推广组织起来就业。强化就业培训，完善和落实各项扶持政策，支持下岗失业人员自主择业、灵活就业和自主创业。要带着感情抓就业，想家长所想，急孩子所急，把帮助青年学生就业放在重要位置。推进就业援助制度化，重点帮助2500名就业困难人员再就业。逐步将城镇集体企业下岗失业人员纳入再就业扶持范围，做好进城求职农民就业服务工作。
　　**进一步完善社会保障体系。**依法推进社会保险扩面征缴，重点做好非公有制经济和灵活就业人员参保工作。建立市级调剂金制度，逐步推进市级统筹。完善城市低保，适当提高低保标准。探索推广农村养老保险试点，建立失地农民保障制度。依法执行最低工资标准，建立防止拖欠农民工工资的长效机制。发展社会慈善事业，做好农村特困救助、优抚安置、社会救济和社会福利等工作，帮助困难群众解决子女上学、看病就医等问题。
　　**加大扶贫开发力度。**加快实施“整村推进”工程，实现整村脱贫170个、脱贫人口15万。加快贫困乡村基础设施建设，加大科技文化培训力度，增强贫困群众自我发展能力。多渠道增加投入，用好扶贫开发资金和以工代赈资金。积极争取库区长效扶持政策，确保扶贫开发的各项政策措施落到实处。
　　**大力加强社会管理。**认真贯彻国务院信访条例，拓宽并畅通信访渠道，进一步落实信访工作责任制和责任追究制。高度重视并认真处理征地、拆迁、企业改制中涉及群众利益的问题，积极预防和妥善处置群体性事件。大力整顿和规范市场经济秩序，扎实开展食品安全监管年活动，严厉打击制假售假和商业欺诈行为。高度重视安全生产，切实开展安全生产大检查和专项整治，坚决防止重特大事故发生。坚持打防结合、预防为主，严厉打击各种违法犯罪行为，推进社会治安防控体系建设，确保社会稳定。

**四、加强政府行政能力建设**

　　坚持以党的十六届四中全会精神为指导，以加强政府行政能力建设为核心，不断增强施政本领。以真心实意对人民负责为宗旨，建设服务型政府；以公开、公平、公正为标准，建设廉洁型政府；以保护和实现公共利益为己任，建设责任型政府；以提高效率、效能为目标，建设绩效型政府；以依法行政为准则，建设法治型政府。
　　**加快转变政府职能，增强驾驭全局的本领。**加强对经济社会发展的战略性研究，科学制定“十一五” 规划和2020年远景目标。创新工作方式方法，善于运用改革的办法和经济、法律的手段解决发展中的矛盾和问题，不断提高发展市场经济的能力。加快政企、政资、政事分开，把政府经济管理职能转到主要为市场主体服务和创造环境上来。加强政务服务中心建设，建立优化环境联席会议制度，强化环境整治持续化、政风评议经常化、案件查处公开化的长效机制。不断完善公共服务体系，积极创新社会管理体制和管理方式，充分发挥基层自治组织、行业和社会中介组织协调利益、提供服务、维护稳定的作用。建立健全社会预警和应急机制，抓紧制定和完善市总体和专项应急预案，不断提高应对突发事件、驾驭复杂局面的能力。推进政府机构改革，深入开展创建学习型、服务型、创新型机关活动，提高行政效能，降低行政成本。
　　**坚持以民为本，增强执政为民的本领。**把人民群众满意作为执政的最高理念，做到重大决策为民而定，措施办法应民而出，工程设施因民而建，安危冷暖唯民而系。坚持深入群众，倾听群众呼声，关心群众冷暖，坚决纠正各种损害群众利益的行为。认真研究和把握新形势下群众工作的特点和规律，善于运用说服教育、示范引导和提供服务等方法把群众工作做深、做细、做实。进一步拓宽新形势下与人民群众联系的渠道，坚持和完善领导干部下访、负责同志接访、定期约访、有访必接和市长热线、市县区长电子信箱等一系列行之有效的制度和措施，让群众有机会说话、有地方诉求、有人帮助解决困难。
　　**推进法制建设，增强依法行政的本领。**认真实施行政许可法和国务院全面推进依法行政实施纲要，在巩固全面清理行政许可规定和实施主体的基础上，切实抓好配套制度建设。完善行政执法体制，相对集中行政处罚权，抓好综合执法试点。强化行政执法监督，严格落实行政执法责任制、评议考核制和执法过错追究制，做到有权必有责、用权受监督、侵权要赔偿。建立和完善重大问题专家咨询、社会公示和听证、决策责任等制度。进一步推进政务公开，完善公开办法，发展电子政务，办好政府网站和政府公报，提高政府工作透明度。各级政府要自觉接受人大法律监督和政协民主监督，认真办理人大议案、代表建议和政协建议案、委员提案。
　　**加强政风建设，增强务实创新的本领。**树立科学发展观和正确政绩观，一切从实际出发，按客观规律办事。坚持讲实话、办实事、求实效，不搞虚报浮夸；坚持统筹兼顾，着眼长远，多干打基础的事，多干长远起作用的事，反对急功近利。进一步改进工作作风，从市政府做起，大力精简会议和文件；提倡开短会、讲短话，开会要解决问题，讲话要有实际内容。围绕重点工作，坚持定期通报和调度制度，强化政务督查，推进工作落实。建设诚信政府，市政府向人代会报告的主要工作必须落实到位，市政府及其部门承诺的事项必须说到做到，不说空话，真办实事。认真落实廉政建设责任制，严肃查处违法违纪案件，坚决纠正部门和行业不正之风。坚持权力与责任紧密挂钩，权力与利益彻底脱钩；坚持政务与阳光同行，行政与腐败分离。进一步规范土地批租、工程招投标、产权交易、政府采购等行为，强化对公共支出、重点工程、专项资金的绩效审计。各级政府要扎实开展保持共产党员先进性教育活动，教育和引导广大公务员树立正确的理想信念和高尚的道德情操，始终保持清正廉洁的正气、敢于争先的锐气、奋发有为的志气，进一步营造风正气顺、凝心聚力、共谋发展的良好氛围。

　　各位代表，回首五年成就，令人鼓舞；展望未来发展，催人奋进。让我们在市委的坚强领导下，围绕第一要务，紧跟时代步伐，弘扬老区传统，坚持与时俱进，抢抓机遇，乘势而上，奋力崛起，为加快发展、富民强市、全面建设小康六安而努力奋斗！
